# Interprofessional collaboration skills and motivation one year after an interprofessional educational intervention for undergraduate medical and nursing students

**DOI:** 10.1186/s12909-024-05262-z

**Published:** 2024-03-11

**Authors:** Carolyn Joyce Teuwen, Rashmi A. Kusurkar, Hermien Schreurs, Hester E. M. Daelmans, Saskia M. Peerdeman

**Affiliations:** 1 Noordwest Academie, Noordwest Ziekenhuisgroep Alkmaar, P.O. box 501, 1800 AM Alkmaar, the Netherlands; 2 Research in Education, Amsterdam UMC Location Vrije Universiteit Amsterdam, De Boelelaan 1118, Amsterdam, the Netherlands; 3 Teaching & Learning Centre (TLC) FdG - UvA, Amsterdam UMC Location AMC, Amsterdam, the Netherlands; 4 LEARN! Research Institute for Learning and Education, Faculty of Psychology and Education, Vrije Universiteit Amsterdam, Amsterdam, the Netherlands; 5 Department of Surgery, Noordwest Ziekenhuisgroep Alkmaar, Alkmaar, the Netherlands; 6 Amsterdam UMC Location Vrije Universiteit Amsterdam, Faculty of Medicine Vrije, Universiteit Amsterdam, Amsterdam, the Netherlands; 7 Department of Neurosurgery, University of Amsterdam, Amsterdam UMC Location Vrije Universiteit Amsterdam, Amsterdam, the Netherlands; 8 Amsterdam Public Health, Quality of Care, Amsterdam, The Netherlands

**Keywords:** Interprofessional education, Interprofessional collaboration, Long-term effect, Motivation, Self-determination theory, Undergraduate medical students, Undergraduate nursing students

## Abstract

**Background:**

The increasingly complex patient care in the twenty-first century is delivered by interprofessional health care teams. Interprofessional collaboration can be taught during interprofessional education. However, whether a long-term change in collaborative competencies can be achieved by interprofessional education has not been studied sufficiently. Our research questions were: How does motivation for interprofessional collaboration and interprofessional collaborative skills change up to one year after an interprofessional educational intervention? How are they related to each other?

**Methods:**

During a one-year period, undergraduate medical and nursing students attended four interprofessional (intervention) or uniprofessional (control group) education sessions. Self-determination Theory was used as the theoretical framework. Autonomous and controlled motivation scores for interprofessional collaboration were calculated using the Academic Self-Regulation Questionnaire, before (T1), directly after (T2) and one year post-intervention (T3). At T3, the students also filled out the Interprofessional Collaborative Competencies Attainment Survey (ICCAS), which measured the perceived attainment of collaborative competencies by a retrospective pre-test/post-test design. We used linear mixed effects models to analyse the motivation scores and linear regression for the relation between motivation and competence.

**Results:**

In the interprofessional group, autonomous motivation scores of the participants were significantly lower at T2 vs. T1. Controlled motivation scores were significantly higher at T3 vs. T1. Controlled motivation scores for T2 were significantly higher in the uniprofessional group than in the interprofessional group. Perceived competence was related to higher autonomous motivation scores. At T3 the interprofessional collaborative competencies seemed to have grown more among students in the interprofessional group.

**Conclusions:**

The perceived growth in interprofessional collaboration competence lasted at least up to one year after the intervention, and was measurable with the ICCAS. The growth was significantly more in the IPE students than in the UPE students. The few differences found in motivation scores for interprofessional collaboration were probably caused by an imbalance of nursing versus medical students over the different time points. This finding indicates that classroom based IPE can contribute to interprofessional collaboration skills of nursing and medical students at least up to one year after an intervention.

## Background

Interprofessional collaboration and communication seems to be inherent to providing good patient care. Nevertheless, miscommunication in health care happens often and leads to medical errors or financial losses [1, 2]. During the Covid-19 pandemic, interprofessional collaboration has proven to be essential to deliver effective high quality care [3].

Also in the healthcare of the future, interprofessional collaboration will become even more important, as the increasingly complex patient care in the twenty-first century can only be delivered by interprofessional health care teams [3]. Throughout the world, many healthcare systems struggle with a shortage of health care workers [4]. Effective interprofessional collaboration can not only help to overcome this shortage, thus helping professionals as well as organisations, but it can also improve patient care [5, 6].

Interprofessional education (IPE) is essential to prepare students for this interprofessional collaboration in clinical practice, but this type of education is often absent in health professions curricula. IPE is defined as “when two or more professions learn with, from and about each other to improve collaborations and the quality of care” [7].

Literature reviews demonstrate that IPE is effective in improving learners attitudes towards other disciplines [8, 9]. IPE may also improve collaborative skills and behaviour. Most studies show positive effects directly after IPE-interventions, such as readiness for interprofessional learning, satisfaction, or attitudes towards other professions. However, more studies are needed that focus on a *long-term* change in collaborative *competencies,* which is the penultimate aim of IPE [8–12].

Outcomes of IPE interventions can be predicted by student motivation [13, 14]. Self-determination Theory (SDT) can help to understand the underlying mechanisms. SDT identifies different kinds of motivation: amotivation, extrinsic and intrinsic motivation. In case of amotivation there is no intention to act. Extrinsic motivated behaviours are driven by external factors, such as to gain a reward or to avoid a negative experience. Extrinsic motivation has different levels of self-determination: external regulation, introjected regulation, identified regulation and integrated regulation. Intrinsic motivation is the most self-determined motivation and makes a person carry out an activity for personal interest. SDT identifies three basic psychological needs, autonomy, relatedness and competence, that need to be fulfilled and stimulate intrinsic motivation. Thus, SDT framework can be used to study the process and effects of interprofessional education [15].

The SDT has not received a lot of attention in the context of interprofessional education. Visser et al. [16] used SDT to study students’ motivation for interprofessional collaboration after an experience on a interprofessional education ward. They found an increase in students’ autonomous motivation for interprofessional collaboration directly after their IPE experience. Ganotice et al. [14, 17] were able to explain variances in behavioural outcomes (e.g. behavioural engagement in an IPE activity) with students’ motivation. The effect of classroom based IPE on students’ motivation and the effect of this motivation on students’ long-term interprofessional collaboration skills has not yet been investigated.

The research questions for the current study were:

- Is there a change in motivation for interprofessional collaboration up to one year after an interprofessional educational intervention?
- Is there a change in perceived interprofessional collaboration skills up to one year after an interprofessional educational intervention?
- Is motivation for interprofessional collaboration associated with interprofessional collaboration competence?

## Methods

### Self-determination theory

**Fig. 1:**
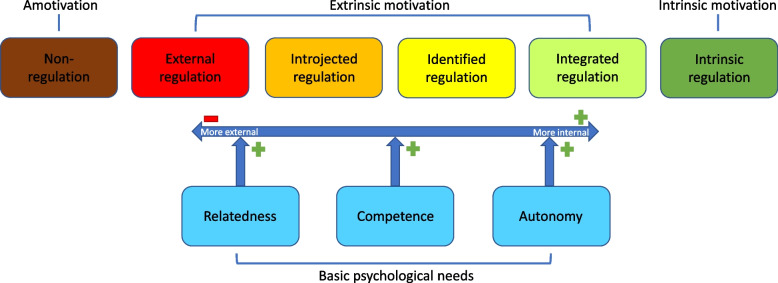
The Self-determination Theory (adapted from Ryan & Deci (2000) [18])

In this study we focused on collaborative competencies and motivation. We used the Self-determination Theory (SDT) framework to study this. Figure 1 depicts the different states of motivation and the influence of the basic psychological needs. Intrinsic motivation, integrated and identified regulation together are often referred to as ‘autonomous motivation’. Introjected and external regulation together are often referred to as ‘controlled motivation’. In this study we used these concepts of ‘autonomous motivation’ and ‘controlled motivation’, since autonomous motivation is associated with more sustained change and better performance [18].

### Participants

Undergraduate medical and nursing students were included. The nursing students were in their third year of a four-year educational program, in which classroom education alternates with participation in clinical practice. Three groups of nursing students (maximum 24 students per group) were asked to participate in this study. The nursing students were individually randomly assigned to the intervention or control group.

The medical students were starting their first year of the master’s program, which consisted of 3 years of learning by participating in healthcare in a range of disciplines during different clerkships, alternated with a few weeks of classroom based teaching. Every six weeks a group of maximum nine students started their clerkships at our educational facility. All groups that started between March 2018 and March 2019 were asked to participate in the study and were assigned as a group to the intervention or control group based on their schedule and if that could match with one of the nursing groups.

Students had no prior interprofessional education experience.

### Setting and assignment

At the Northwest Clinics in Alkmaar, the Netherlands, students were asked to draw up health care plans for paper-based geriatric patient cases, four times over a one-year period. The four cases, with an increasing level of difficulty during the year and typical geriatric care problems, were constructed through discussion with different geriatric experts [19].

In the control group, students wrote the health care plans on their own (uniprofessional education group, i.e. UPE-group). In the intervention group, the health care plans were written by randomly paired medical and nursing students (interprofessional education group, i.e. IPE-group). In each session different pairs were assembled to create diversity among the collaboration partners. Each session lasted for one hour.

### Questionnaires

To assess the students’ motivation for interprofessional collaboration, a Dutch version of the Academic Self-Regulation Questionnaire (SRQ-A) was used [20]. This 16-item questionnaire measures individual differences in the four types of regulation: external regulation, introjected regulation, identified regulation and intrinsic motivation. Each of the 16 items was scored on a 5-point Likert scale. A score for autonomous motivation was calculated as an average of intrinsic motivation and identified regulation scores. Controlled motivation was calculated as an average of introjected and external regulation scores. Validity and reliability of the measurement of motivation with the Dutch version of the SRQ-A have been reported in earlier studies, including its suitability for measurement among medical students [21–23].

The Interprofessional Collaborative Competencies Attainment Survey (ICCAS) was used to assess the change in the students’ interprofessional collaboration-related competencies after the intervention. The ICCAS is validated for such purpose with undergraduate students [24, 25]. The ICCAS uses a retrospective pre-test/post-test design to the self-assessment, which means that the participants only fill out the questionnaire once after the intervention and rate their ability for each statement twice: Once for ‘pre’ (before the intervention) and once for ‘post’ (after the intervention, in our study one year after the intervention, see Fig. 1). The twenty ICCAS items are related to interprofessional communication, collaboration, roles and responsibilities, collaborative patient-family-centered approach, conflict management/resolution, and team functioning, and are answered on a 1–7 Likert-scale. As recommended by Schmitz et al. [25] and Lunde et al. [26] we used 1 overall score ‘pre’ and 1 overall score ‘post’ of the ICCAS and did not analyse all separate items. The overall scores are calculated as the means of all items ‘pre’ and all items ‘post’. The ICCAS was translated from English into Dutch by using the Beaton translation method [27].

**Fig. 2:**
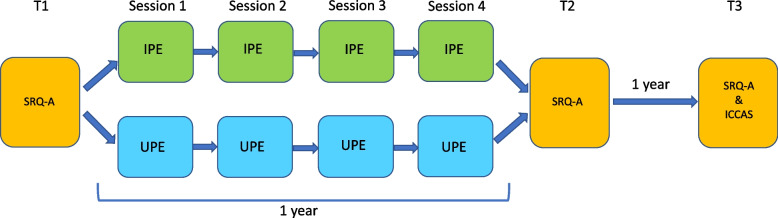
Research questionnaires and assignments timeline

Before (T1), directly after (T2) and one year after the intervention (T3) the students filled out the SRQ-A. At T3, the students also filled out the ICCAS. At T1 and T2 students were present in the classroom to fill out the questionnaires, at T3 the questionnaires were sent by email. Figure 2 depicts the research timeline.

### Statistical analysis

SPSS version 28.0.0.0 was used to process and analyse the results.

Missing data were imputed with the ‘average of the available items’: Within one participant, the scores of the questions that refer to the same motivation type as the missing value, were used to calculate an average. This average score replaced the missing value.

We used linear mixed effects models with random intercept and fixed effects for group (uniprofessional versus interprofessional) and time, to assess the longitudinal change in autonomous motivation scores and controlled motivation scores within each group. Timepoints were included as factor to account for non-linear effects over time. To study autonomous and controlled motivation scores over time within the interprofessional group, we repeated the linear mixed model analyses with interprofessional participants as reference category. Interaction effects between group and time were used to assess whether autonomous motivation scores and controlled motivation scores trajectories differed over time between the uniprofessional and interprofessional participants. Simple linear regression analysis was performed to investigate the relationship between autonomous motivation at T3 (predictor) and the ICCAS post-score (dependent variable).

A power analysis was conducted for both the variables motivation and skills to determine the sample size. With an anticipated mean of 3.5 for motivation for the UPE-group and 4.0 (SD ± 0.5) for the IPE group, alpha = 0.05 and a power of 0.8, the calculated sample size was 16 participants per group. An anticipated mean of 4 for interprofessional collaboration skills for the UPE-group and 5 (SD ± 1) for the IPE group, alpha = 0.05 and a power of 0.8, the calculated sample size also was 16 participants per group. For the increase score of the ICCAS we anticipated a mean of 0.5 for the UPE-group and 1 (SD ± 0.2) for the IPE group, alpha = 0.05 and a power of 0.8. The calculated sample size for this analysis was 3 participants per group.

## Results

### Group characteristics

A total number of 127 students were included in the study. In both, UPE and IPE groups, the majority was female, which reflects the gender distribution in both medical (≈70%) and nursing curricula (≈90%) in the Netherlands [28, 29]. Gender, age and the proportion of nursing students versus medical students was not statistically different between both groups (Table 1).

**Table 1:** Group characteristics at T1

	Uniprofessional Education *N* = 68	Interprofessional Education *N* = 61	*p*-value
*Nursing students n(%)*	28 (41%)	30 (49%)	0.381
*Female n(%)*	57 (84%)	43 (71%)	0.091
*Age, years (± sd)*	21.8 (± 2.7)	21.0 (± 2.6)	0.080
*Prior IPE experience n(%)*	5 (7%)	4 (6%)	1.000

% Nursing students, % Female, % prior IPE experience: Fisher’s exact

Age: Independent samples T-test

At T2 and T3 not all included students filled out the SRQ-A and at T3 not all students filled out the ICCAS. At T2 students received the questionnaire in the classroom, but at two session the questionnaire was not handed out or students forgot to fill it out (missing *n* = 25). At T3 the questionnaires were sent by email. Despite multiple reminders, not all students responded to the mail (missing *n* = 68). It could be that some students at T2 or T3 had already quit their educational program, but because of privacy reasons we were not kept informed about their personal circumstances.

**Fig. 3:**
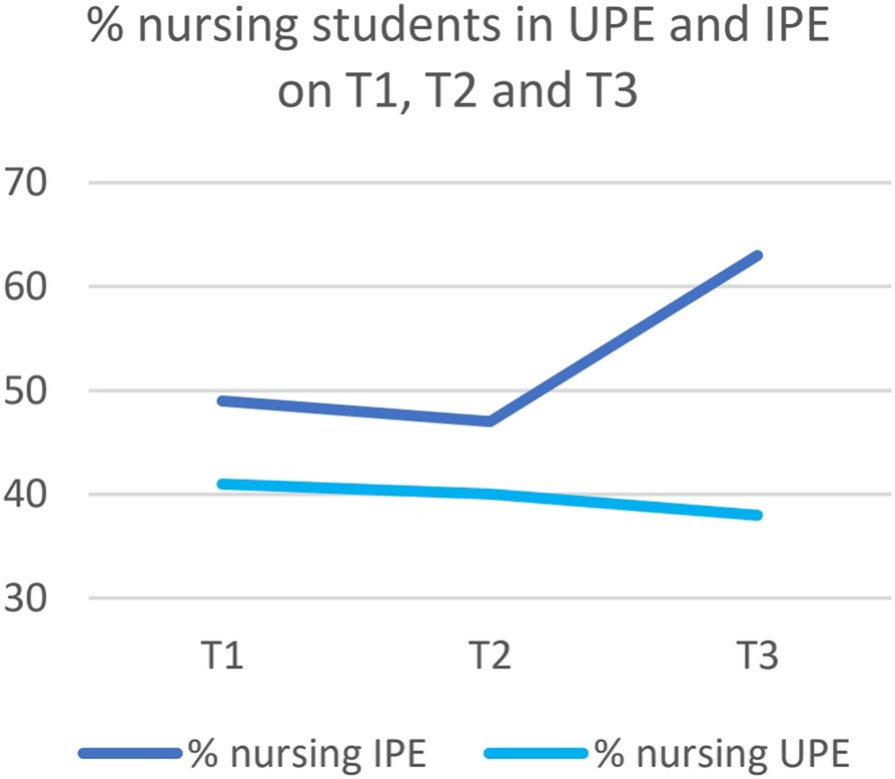
Proportion (%) of nursing students in the IPE and UPE groups at T1, T2 and T3

The proportion of nursing versus medical students that responded to the questionnaires at T3, between the UPE and IPE groups differed (Fig. 3).

### Academic self-regulation questionnaire

The descriptive results of the Academic Self-regulation questionnaire are shown in Table 2.

**Table 2:** Controlled and autonomous motivation for interprofessional collaboration in the UPE and IPE group at T1, T2 and T3

	**T1**		**T2**		**T3**	
	***n*(%)**	**Mean ± SD**	***n*(%)**	**Mean ± SD**	***n*(%)**	**Mean ± SD**
**UPE**	67(100%)		45(67%)		32(48%)	
Autonomous motivation		3.9 ± 0.58		3.8 ± 0.55		3.8 ± 0.62
Controlled motivation		2.3 ± 0.64		2.5 ± 0.69		2.5 ± 0.68
**IPE**	60(100%)		57(95%)		27(45%)	
Autonomous motivation		4.0 ± 0.48		3.8 ± 0.58		4.0 ± 0.58
Controlled motivation		2.4 ± 0.76		2.2 ± 0.74		2.9 ± 0.84

Motivation scores: Likert 1–5

In the UPE-group, the autonomous and controlled motivation for interprofessional collaboration scores did not change significantly over time.

In the IPE-group, autonomous motivation scores were significantly lower on T2 vs. T1 (β = -0.21 95%CI [-0.42: -0.01], *p* = 0.041) and controlled motivation scores were significantly higher on T3 vs. T1 (β = 0.43 95%CI [0.15: 0.81], *p* = 0.004).

**Fig. 4:**
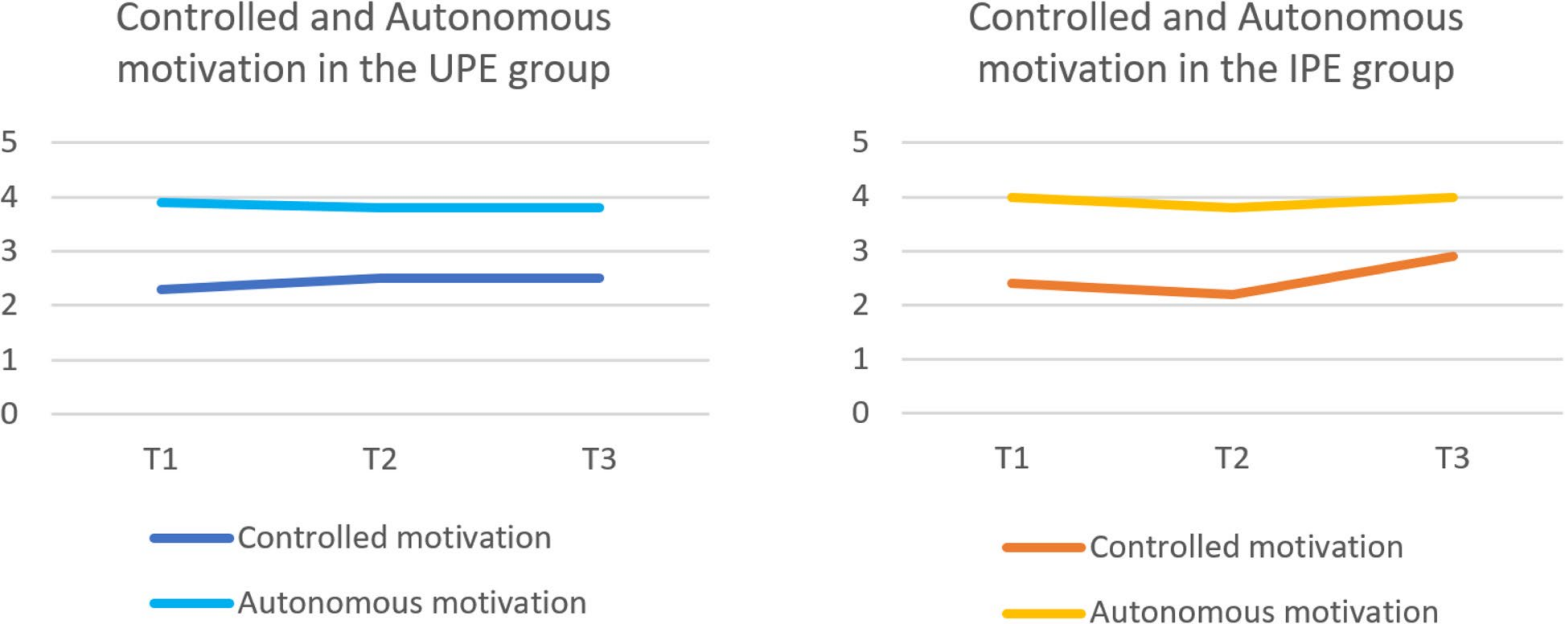
Controlled and autonomous motivation for interprofessional collaboration in the UPE and IPE groups

When comparing the UPE-group with the IPE group, the trajectory of the controlled motivation scores over time was significantly higher at T2 in the UPE students than in the IPE students. All others scores between the groups were not significantly different (Table 3, Fig. 4).

**Table 3:** Differences between the autonomous en controlled motivations scores compared to T1 in each group

	**T2 vs T1**			**T3 vs T1**		
	***n*(%)**	**Beta [95%CI]**	***p*-value**	***n*(%)**	**Beta [95%CI]**	***p*-value**
**UPE**	45(67%)			45(67%)		
Autonomous motivation		-.09 [-.31 – .12]	0.391		-.15 [-.38 – .09]	0.223
Controlled motivation		.23 [-.04 – .50]	0.097		.21 [-.09 – .51]	0.175
**IPE**	57(95%)			57(95%)		
Autonomous motivation		-.21 [-.42 – -.01]	0.041		-.01 [-.27 – .25]	0.940
Controlled motivation		-.23 [-.49 – .03]	0.085		.48 [.15 – .81]	0.004

Motivation scores: Likert 1–5

#### ICCAS

The response rate of the ICCAS was 46% (*n* = 59). The age of the UPE-group was significantly higher (22 versus 20.5 years old) than that of the IPE-group. Gender and the proportion of nursing students was not significantly different between the groups. The mean pre and mean post scores of the ICCAS did not differ between the UPE and IPE groups. The difference between the pre and post score was significantly different between the UPE and IPE groups, with an moderate effect size (Cohen’s D: 0.588). Educational level (nursing vs medical), gender and age were not associated with differences in ICCAS scores between the groups. Table 4 depicts these results.

**Table 4:** Comparison of student characteristics at T3 and ICCAS scores of the UPE and IPE groups

	UPE (*n* = 32)	IPE (*n* = 27)	Sig. *p* =
*Nursing students n(%)*	12 (38%)	17 (63%)	0.069
*Female n(%)*	30 (94%)	20 (74%)	0.066
*Age, years (*± *sd)*	22 (± 2.9)	20.5 (± 2.8)	0.046*
*Mean pre, mean (*± *sd)*	5.1 (± 1.1)	4.8 (± 1.0)	0.190
*Mean post, mean (*± *sd)*	5.8 (± 0.8)	6.0 (± 0.7)	0.296
*Mean difference, mean (*± *sd)*	0.7 (± 0.9)	1.3 (± 1.1)	0.028*

ICCAS: Likert 1–7

% Nursing students, % Female: Fisher’s exact

Age, Mean pre, Mean post, Mean difference: Independent samples T-test

^*^p < 0.05

### Association SRQ-A and ICCAS

The regression analysis showed a statistically significant association between the post-score at the ICCAS and autonomous motivation at T3 (*R*^2^ = 0.247, *F* = 19,073, *p* < 0.001). The fitted regression model was: *y* = *3.56* + *0.611x* (*p* < 0.001). A higher autonomous motivation for interprofessional collaboration at T3 (predictor) was associated with a higher post-score at the ICCAS (dependent variable).

## Discussion

In this study we looked at the effect of IPE on motivation for interprofessional collaboration and interprofessional collaborative skills of undergraduate nursing and medical students one year after the intervention. We also studied the association between motivation and collaborative skills in this context. We found a significant relationship between interprofessional collaborative skills and autonomous motivation for interprofessional collaboration at T3. The more motivated nursing and medical students are to collaborate interprofessionally, the more competent they are at those skills. This is in line with the Self-determination Theory, in which autonomous motivation is associated with more sustained change and better performance [18]. This finding implicates that stimulating the autonomous motivation of students to collaborate interprofessionally, will result in better interprofessional collaborative skills. According to SDT, stimulation of the autonomous motivation can be achieved by fulfilling the basic psychological needs of autonomy, belongingness and competence. In interprofessional education, autonomy could be fulfilled, for example, by letting students decide what kind of interprofessional activity they participate in, to practice their skills. While practicing interprofessional skills, students’ feeling of competence will also grow, fulfilling the second basic psychological need. The feeling of belonginess could be achieved by more contact and joint activities between students of different educational programs, such as medicine, nursing, and pharmacy. For example, interprofessional education wards could also facilitate this [16].

Although we expected that the IPE intervention would stimulate students autonomous motivation, despite an adequate sample size, we did not find that in our results. This could be caused by a ceiling effect, since autonomous motivation scores in the IPE-group were already 4.0 at T1. However, with a mean score of 4.0 at T3, we could still say that the IPE-students in our study were autonomously motivated to collaborate interprofessionally, but maybe the intervention was not able to increase this motivation.

Most motivation scores were not significantly different in between the different timepoints or between the groups, but some significant differences were found. The IPE-students had significantly less autonomous motivation directly after the intervention (T2) compared to their scores at T1. And, compared to the UPE-group, the IPE-group had also lower controlled motivation scores at T2. We were unable to explain these two findings based on the nature of the intervention. However, the bigger proportion of nursing students in the IPE-group could explain the change and difference in controlled as well as in autonomous motivation. If their motivation is different than that of the medical students, the motivation scores will change according to the change in proportions. The motivation in nursing students could be different because the curricula of medical and nursing students in our study differ. The nursing students in our study worked on the ward four days a week, collaborating with different kinds of professionals. This is in contrast to the medical students in this study, who often stated that they only collaborated with their own profession during their internships. Because the nursing students didn’t have a choice and collaborating interprofessionally was incorporated in their curriculum, that could have contributed to more controlled motivation. Meanwhile, this experience in clinical practice could make nursing students also more autonomously motivated, as seeing the added value of interprofessional collaboration themselves. The different opinions of nurses and physicians and their interprofessional collaboration has been described before in the literature. In a review study by Tang et al. [30] nurses also seem to appreciate collaboration with physicians more than vice versa.

Our last finding in this study concerns the interprofessional collaborative competencies of the nursing and medical students. The students in the IPE-group in our study perceived to have grown more in their interprofessional competencies than the students in the UPE-group. The ‘after-scores’ (T3) were not significantly different between the IPE and UPE groups, but IPE-students rated themselves somewhat lower in the pre-score and somewhat higher in the after-score than UPE-students. Maybe IPE-students realized what they did not know about interprofessional collaboration *because* the attended IPE-sessions. They became consciously incompetent instead of unconsciously incompetent. It is an interesting finding that our intervention was big enough to achieve such a difference and also that the difference is still significant one year after the intervention. This adds to the literature of the long-term effects of IPE-interventions, since such a finding has not been described before. A few other studies have also used the ICCAS after an IPE-intervention and they have reported a positive change between pre and post scores, but this was measured directly after the intervention [31–33]. Gunaldo et al. [34] did study the long-term effect of a one-time IPE-intervention on interprofessional collaboration using the ICCAS, one year after the intervention. They did not find significant change, but this may have been because their intervention was shorter than ours. Mink et al. [35] used another questionnaire, the Assessment of Interprofessional Team Collaboration Scale and found an increase of perceived interprofessional skills directly after, but also 3 months after the IPE-intervention. The effect size of 0.6 measured 3 months after the intervention was similar to the effect size we found in our study. McNaughton et al. [11], in their scoping review, describe that most studies tend to have positive long-term effects of IPE on interprofessional collaboration, but that most studies use self-measurements. This is also a limitation in our study.

Our study was subject to some limitations. The response at T3 was relatively low, and, compared to the UPE-group, the proportion of nursing students in the IPE-group was bigger at T3. Although the sample size was adequate, this could have influenced the results. Future research with larger sample sizes could find significant differences between nursing and medical students. Also more different professions could be included, such as pharmacy and physical therapy. The second limitation is the self-measurement tool we used, the ICCAS. Although we found significant differences between the intervention and the controlled group, the outcome is *perceived* attainment of competencies, not the actual performance in clinical practice. Data collection of the ICCAS at T2 could have helped to determine competency development. This is a limitation of our study, since we did not collected this data. Collecting the ICCAS data at several points in time would be an implication for future research involving the ICCAS [36]. In addition, it would be interesting to know, if students' acquired competencies are carried beyond graduation into early interprofessional practice. Measuring their’ skills just after graduation could clarify this pattern.

Finally, future research should focus on finding tools to measure actual interprofessional competencies of students in clinical practice. The ICCAS maybe suitable for this, but other methods can also be considered, such as a 360-degree feedback.

## Conclusions

In this study we looked at the long-term effect of IPE on motivation for interprofessional collaboration and perceived competence for interprofessional collaborative skills. More autonomous motivation for interprofessional collaboration was associated with more interprofessional competence. The perceived growth in interprofessional collaboration competence lasted at least up to one year after the intervention, and was measurable with the ICCAS. The growth was bigger in the IPE students than in the UPE students. This finding indicates that classroom based IPE, if offered to a sufficient extent, can contribute to interprofessional collaboration skills of nursing and medical students in the long term.

## Data Availability

The datasets generated and analysed during the current study are not publicly available due to ethical considerations. The privacy officers of Amsterdam UMC advised us to not share the data, because in the informed consent form, we did not specifically take the participants’ permission for openly sharing their data. We are not able to do it retroactively. We anonymized the file, but because it is a small sample size, and we included two different groups of students (medical and nursing), students (or others) might be able to identify themselves. No intermediary data can be de-identified without compromising anonymity. Thus, our data cannot be shared with others. The corresponding author can be contacted for questions about the data and the data availability, at c.j.teuwen@nwz.nl.

## Abbreviations

IPE: Interprofessional education
UPE: Uniprofessional education
SRQ-A: Academic self-regulation questionnaire
SDT: Self-determination theory
ICCAS: Interprofessional Collaborative Competencies Attainment Survey
Sd: Standard deviation

## References

[1] Clapper TC, Ching K (2019). Debunking the myth that the majority of medical errors are attributed to communication. Med Educ.

[2] Agarwal R, Sands DZ, Schneider JD (2010). Quantifying the economic impact of communication inefficiencies in US hospitals. J Healthc Manag.

[3] Hennus MP, Young JQ, Hennessy M, Friedman KA, de Vries B, Hoff RG (2021). Supervision, Interprofessional Collaboration, and Patient Safety in Intensive Care Units during the COVID-19 Pandemic. ATS Sch.

[4] Boniol M, Kunjumen T, Nair TS, Siyam A, Campbell J, Diallo K (2022). The global health workforce stock and distribution in 2020 and 2030: a threat to equity and ‘universal’ health coverage?. BMJ Glob Health.

[5] Wei H, Horns P, Sears SF, Huang K, Smith CM, Wei TL (2022). A systematic meta-review of systematic reviews about interprofessional collaboration: facilitators, barriers, and outcomes. J Interprof Care.

[6] Gilbert J, Yan J, Hoffman SJ (2010). A WHO report: Framework for Action on Interprofessional Education & Collaborative Practice. J Allied Health.

[7] Barr H (2002). CAIPE 2002 Interprofessional Education, Today, Yesterday and Tomorrow - A review.

[8] Spaulding EM, Marvel FA, Jacob E, Rahman A, Hansen BR, Hanyok LA (2021). Interprofessional education and collaboration among healthcare students and professionals: a systematic review and call for action. J Interprof Care.

[9] Reeves S, Fletcher S, Barr H, Birch I, Boet S, Davies N (2016). A BEME systematic review of the effects of interprofessional education: BEME Guide No 39. Med Teach.

[10] Marion-Martins AD, Pinho DLM (2019). Interprofessional simulation effects for healthcare students: A systematic review and meta-analysis. Nurse Educ Today.

[11] McNaughton S (2018). The long-term impact of undergraduate interprofessional education on graduate interprofessional practice: A scoping review. J Interprof Care.

[12] Rodrigues da Silva Noll Gonçalves J, Noll Gonçalves R, SV da Rosa, Schaia Rocha Orsi J, Santos de Paula KM, SJ Moysés (2023). Potentialities and limitations of Interprofessional Education during graduation: a systematic review and thematic synthesis of qualitative studies. BMC Med Educ.

[13] Ganotice FA, Chan CS, Chan EWY, Chan SKW, Chan L, Chan SCS (2022). Autonomous motivation predicts students' engagement and disaffection in interprofessional education: Scale adaptation and application. Nurse Educ Today.

[14] Ganotice FA, Gill H, Fung JTC, Wong JKT, Tipoe GL (2020). Autonomous motivation explains interprofessional education outcomes. Med Educ.

[15] Ganotice FA, Chan KMK, ChanSL,ChanSsC, Fan KKH, Lam MPS, (2023). Applying motivational framework in medical education: a self determination theory perspectives. Med Educ Online.

[16] Visser CLF, Kusurkar RA, Croiset G, ten Cate O, Westerveld HE (2019). Students’ motivation for interprofessional collaboration after their experience on an IPE ward: A qualitative analysis framed by self-determination theory. Med Teach.

[17] Ganotice FAJ, Chan L, Chow AYM, Khoo US, Lam MPS, Liu RKW (2022). What characterize high and low achieving teams in Interprofessional education: A self-determination theory perspective. Nurse Educ Today.

[18] Ryan RM, Deci EL (2000). Self-Determination Theory and the Facilitation of Intrinsic Motivation, Social Development, and Well-Being. Am Psychol.

[19] Teuwen CJ, Kusurkar RA, Schreurs WH, Daelmans HE, Peerdeman SM (2020). The Validation of Geriatric Cases for Interprofessional Education: A Consensus Method. J Med Educ Curric Dev.

[20] Ryan RM, Connell JP (1989). Perceived locus of causality and internalization: Examining reasons for acting in two domains. J Pers Soc Psychol.

[21] Wouters A, Croiset G, Schripsema NR, Cohen-Schotanus J, Spaai GWG, Hulsman RL (2017). A multi-site study on medical school selection, performance, motivation and engagement. Adv in Health Sci Educ.

[22] Isik U, Wouters A, Ter Wee MM, Croiset G, Kusurkar RA (2017). Motivation and academic performance of medical students from ethnic minorities and majority: a comparative study. BMC Med Educ.

[23] Vansteenkiste M, Sierens E, Soenens B, Luyckx K, Lens W (2009). Motivational profiles from a self-determination perspective: The quality of motivation matters. J Educ Psychol.

[24] Archibald D, Trumpower D, MacDonald CJ (2014). Validation of the interprofessional collaborative competency attainment survey (ICCAS). J Interprof Care.

[25] Schmitz CC, Radosevich DM, Jardine P, MacDonald CJ, Trumpower D, Archibald D (2017). The Interprofessional Collaborative Competency Attainment Survey (ICCAS): A replication validation study. J Interprof Care.

[26] Lunde L, Bærheim A, Johannessen A, Aase I, Almendingen K, Andersen IA (2021). Evidence of validity for the Norwegian version of the interprofessional collaborative competency attainment survey (ICCAS). J Interprof Care.

[27] Beaton DE, Bombardier C, Guillemin F, Ferraz MB (2000). Guidelines for the Process of Cross-Cultural Adaptation of Self-Report Measures. Spine (Philadelphia, Pa. 1976).

[28] Opnieuw willen minder mensen geneeskunde studeren. Available at: https://www.medischcontact.nl/actueel/laatste-nieuws/nieuwsartikel/opnieuw-willen-minder-mensen-geneeskunde-studeren. Accessed 28 Nov 2023.

[29] Weinig mannen kiezen voor een zorgopleiding. Available at: https://www.cbs.nl/nl-nl/nieuws/2016/11/weinig-mannen-kiezen-voor-een-zorgopleiding. Accessed 28 Nov 2023.

[30] Tang CJ, Chan SW, Zhou WT, Liaw SY (2013). Collaboration between hospital physicians and nurses: An integrated literature review. Int Nurs Rev.

[31] Guy JW, Claus E, Witsken C, Oestreich JH (2022). Delivery of interprofessional education through a co-curricular journal reviewing medical literature. Curr Pharm Teach Learn.

[32] Kruger JS, Tona J, Kruger DJ, Jackson JB, Ohtake PJ (2023). Validation of the Interprofessional Collaborative Competency Attainment Survey (ICCAS) retrospective pre-test measures. J Interprof Care.

[33] King J, Beanlands S, Fiset V, Chartrand L, Clarke S, Findlay T (2016). Using interprofessional simulation to improve collaborative competences for nursing, physiotherapy, and respiratory therapy students. J Interprof Care.

[34] Gunaldo T, Rosenbaum C, Davis A (2021). Long-term impact of a single interprofessional education high-fidelity simulation experience: a pilot study. BMJ Simul Technol Enhanc Learn.

[35] Mink J, Mitzkat A, Krug K, Mihaljevic A, Trierweiler-Hauke B, Götsch B (2021). Impact of an interprofessional training ward on interprofessional competencies – a quantitative longitudinal study. J Interprof Care.

[36] Violato EM, King S (2019). A Validity Study of the Interprofessional Collaborative Competency Attainment Survey: An Interprofessional Collaborative Competency Measure. J Nurs Educ.

